# Socioeconomic Correlates of Eating Disorder Symptoms in an Australian Population-Based Sample

**DOI:** 10.1371/journal.pone.0170603

**Published:** 2017-01-31

**Authors:** Brittany Mulders-Jones, Deborah Mitchison, Federico Girosi, Phillipa Hay

**Affiliations:** 1 School of Medicine, Western Sydney University, Sydney, NSW, Australia; 2 Centre for Emotional Health, Macquarie University, Sydney, NSW, Australia; 3 Centre for Health Research, Western Sydney University, Sydney, NSW, Australia; 4 Capital Markets CRC, Sydney, NSW, Australia; Hospital Universitari de Bellvitge, SPAIN

## Abstract

**Background:**

Recent research has challenged the stereotype that eating disorders are largely limited to young, White, upper-class females. This study investigated the association between indicators of socioeconomic status and eating disorder features.

**Methods and Findings:**

Data were merged from cross-sectional general population surveys of adults in South Australia in 2008 (*n* = 3034) and 2009 (*n* = 3007) to give a total sample of 6041 participants. Multivariate logistic regressions were employed to test associations between indicators of socioeconomic status (household income, educational level, employment status, indigenous status and urbanicity) and current eating disorder features (objective binge eating, subjective binge eating, purging, strict dieting and overvaluation of weight/shape). Eating disorder features occurred at similar rates across all levels of income, education, indigenous status, and urbanicity (*p* > 0.05). However, compared to working full-time, not working due to disability was associated with an increased risk of objective binge eating (odds ratio (OR) = 2.30, *p* < 0.01) and purging (OR = 4.13, *p* < 0.05), engagement in home-duties with an increased risk of overvaluation of weight/shape (OR = 1.39, *p* < 0.05), and unemployment with an increased risk of objective binge eating (OR = 2.02, *p* < 0.05) and subjective binge eating (OR = 2.80, *p* < 0.05). Furthermore, participants with a trade or certificate qualification were at a significantly increased risk of reporting strict dieting compared to participants without a tertiary qualification (OR = 1.58, *p* <0.01). Limitations included the small numbers of indigenous participants (*n* = 115) and participants who reported purging (*n* = 54), exclusion of excessive exercise (which is associated with eating disorders, particularly in males), and the conduct of interviews by laypersons.

**Conclusions:**

Overall, symptoms of eating disorders are distributed equally across levels of socioeconomic status. This study highlights the need for universal access to specialised services, to train healthcare workers in the detection and diagnosis of eating disorders in diverse subgroups, and to combat barriers to help-seeking experienced by people who do not conform to the demographic stereotype of an eating disorder. The increased prevalence of various eating disorder features in those who are not working could be addressed by providing support to help sufferers join the workforce, or engage in meaningful social or community activities to improve resilience against the development of eating disorders.

## Introduction

Eating disorders (EDs) are characterised by disturbances in eating and feeding behaviours with accompanying psychological concerns that pertain to eating and body image. The primary clinical entities outlined in the fifth edition of the Diagnostic and Statistical Manual of Mental Disorders are anorexia nervosa (AN), bulimia nervosa (BN) and binge eating disorder (BED) [1].

Prominent theoretical models posit overvaluation of body weight/shape (i.e. assigning excessive importance to weight and shape in evaluating self-worth) as trans-diagnostic across AN, BN, and BED and core to the maintenance of behavioural eating disorder symptoms [2]. Extreme weight control behaviours such as caloric restriction or purging (e.g., self-induced vomiting, laxative misuse) are observed in AN and BN but not in BED [1]. Conversely objective binge eating (i.e., feeling a sense of loss of control whilst eating an unusually large amount of food) is a core symptom of both BN and BED, but may also be present in AN [1]. Subjective binge eating is another common trans-diagnostic problematic behaviour [1]. It is defined as experiencing a sense of loss of control whilst eating small or normal amounts of food [1]. People with disordered eating patterns that incorporate these symptoms but fail to meet full diagnostic criteria for AN, BN, or BED may be diagnosed with the category “Other Specified or Unspecified Eating Disorders” (OSFED or UFED) [1].

The estimated lifetime prevalence of EDs range from 1.0% (AN) to 3.5% (BED) in women and < 0.5% (AN) to 2.0% (BED) in men [3]. The prevalence of OSFED and UFED is yet to be comprehensively investigated. However, Allen et al. found that, in adolescents at least, these disorders account for 15–40% of all ED cases [4]. EDs confer significant cost both to the individual and society. AN has the highest mortality rate of any psychiatric illness [5]. Furthermore, there is an extremely high prevalence of comorbid psychiatric disorders amongst all three major EDs. For instance, in the National Comorbidity Replication Study, Hudson et al. found that the vast majority of ED sufferers (54.2% of AN, 94.5% of BN, and 78.9% of BED) also met criteria for one or more other mental disorders, including mood, anxiety, impulse-control, and substance use disorders [6]. Worldwide burden and fiscal costs of EDs are high with yearly estimates of €2993 to €55 270, €888 to €18 823, and €1762 to €2902 for treatment of AN, BN and BED respectively [7]. Furthermore, a report by Deloitte Economics concluded that the Australian annual “burden of disease” costs for EDs ($52.5 billion) were comparable to obesity ($52.9 billion) and exceeded those of anxiety and depression combined ($41.2 billion) [8].

As a result of the historical belief that EDs were diseases of wealthy, White, young females [9], the provision of treatment has neglected other demographic groups which research has since shown to be affected by disordered eating. Such groups include males [6], middle-aged and older people, and people of non-White ethnicity [10, 11]. For instance, Becker et al. found that Latino and Native American people with self-reported disordered eating were significantly less likely to have been asked about ED features by their general practitioner than European American women [12]. Furthermore, women of ethnic minorities were also less likely to seek treatment. The same study similarly found low rates of treatment seeking in males, with only 29.1% of male participants with BN and 28.9% with BED seeking treatment for their ED in their lifetime compared to 47.0% and 50.8% respectively amongst female participants. In a review pooling treatment-seeking estimates, these rates are even lower, with less than one in four people with EDs receiving professional help [13]. Another widespread issue is the overreliance of past research on clinical rather than community samples [14]. As the majority of people who experience eating disorders are not treatment-seekers [13], a treatment-seeking sample is unlikely to be representative of people with eating disorders in the population. Further, clinical samples are usually disproportionately representative of diagnoses such as AN and BN, compared to more prevalent conditions such as BED, OSFED and UFED.

It is thus clear that EDs are not limited to young metropolitan females and it is pertinent to determine exact distribution of disordered eating in order to provide all sufferers with adequate and equal access to treatment. This requires the use of epidemiological studies using community and population-based samples, free from selection bias. In their analysis of two sequential cross-sectional surveys of South Australian residents conducted in 1998 and 2008, Mitchison et al. found the prevalence of ED features (objective binge eating, purging and extreme dieting) in participants of below-median household income to be comparable to or even greater than in participants of above-median income [15]. Additionally, comparison of the two survey years showed that the prevalence of these features increased at a greater rate in the below-median group. Swanson et al. likewise found no association between household income and any ED [16]. These studies, though important in calling long-held assumptions regarding the socioeconomic correlates of EDs into question, are limited in their methodologies. In the study by Mitchison et al. [15], the division of income into two brackets, above and below the median, is crude and more precise categorisation is needed to determine the level at which SES becomes associated with increased risk of ED features. Further, the study by Swanson et al. included only adolescent participants [16] and its findings can thus not be generalised to the broader adult population.

Studies evaluating the impact of other correlates such as educational attainment, employment status, indigenous origin and urbanicity provide a more holistic assessment of the association between SES and ED prevalence. Research thus far that has examined the association between educational attainment and ED prevalence has yielded mixed results. For example, AN has been associated with characteristics such as perfectionism, a strong work ethic and high academic performance [17, 18]. On the contrary, impaired concentration may result from starvation or comorbid depression and lead to poorer educational outcomes [19]. Mitchison and Hay’s systematic review of 149 papers failed to support an association between educational attainment and ED prevalence [20]. The majority of studies assessing this particular correlate showed no relationship; of the remaining studies, lower and higher education were equally found to be associated with increased prevalence of EDs. This inconsistency in the research literature may stem from methodological differences such as investigation of AN only or a range of EDs, and necessitates further investigation to confirm the direction of any correlation.

Although unemployment has frequently been associated with a number of psychiatric disorders [21, 22], the association between employment status and ED prevalence has been largely neglected in research. At the time of writing, the only existing study is a cross-sectional survey of college students in Greece [23]. Students were separated into two groups based on their scores on a brief screening measure [24]: those at risk for AN or BN, and those not considered at risk. The study found no significant effect of employment status on ED risk. There are, however, some significant limitations to this study. As the sample included only college students, the results cannot be generalised to the wider community. Furthermore, with all participants being students, employment status categories were limited to comparing students who were not working with those working either part- or full-time, which represents only a very small portion of the employment spectrum. Finally, this study does not acknowledge that undertaking a college education is a meaningful occupation in itself, and thus a more fruitful investigation might compare those who are studying or working to those who are not engaged in any academic or occupational pursuits. It is clear that further research, from a representative sample of the general population, is necessary.

Indigenous Australians have been shown to be disadvantaged relative to their non-indigenous counterparts across many socioeconomic markers, including education, employment, income and access to services [25], and thus indigenous status may be viewed as a correlate of SES. Despite the well-known high prevalence of weight-related health problems in Indigenous Australians [25] and the association between BED and BN, and obesity [6], little research has been conducted to investigate disordered eating in this group. At the time of writing, there is only one study on this topic: an epidemiological study [26] conducted using data from two surveys of the South Australian population, the first collected in 2005 and the second in 2008. Hay and Carriage found that ED features–binge eating, overvaluation of body weight/shape, extreme dieting and compensatory weight control behaviours–occurred in indigenous persons at a rate similar to that of non-indigenous Australians after controlling for age, body weight and income levels. Although this study is limited by the small number of indigenous participants in the population sample, it indicates that persons of indigenous origin are certainly not exempt from being afflicted with EDs.

Data regarding the effect of urbanicity on the prevalence of EDs is also particularly lacking. A Dutch epidemiological study [27] assessed the difference in incidence of AN and BN between cities, urbanised areas and rural areas. While no significant difference in the incidence of AN between these locations was found, BN was significantly more common in more urbanised areas (37.9 per 100 000 in cities, 19.9 in urban areas and 6.6 in rural areas). This study, however, is limited in that it only included participants registered by their general practitioner as having a diagnosis of AN and/or BN. As outlined previously, clinical samples such as this cannot be assumed to be representative of the population with EDs. A number of other studies have shown no significant differences in the prevalence of EDs or ED behaviours between regional and metropolitan areas [15, 16]. These studies, however, are limited by their simple division of urbanicity into metropolitan versus rural. More specific grading of urbanicity is necessary to confirm what has been suggested by these studies, and to indicate the level of rurality or urbanicity associated with particular ED features. Furthermore, it is unclear as to whether any effects of rurality found were due to remoteness per se–including lack of access to services–or rather due to lower income levels associated with less urban areas. Analysing both urbanicity and income in a single study sample could help to determine the degree to which each factor accounts for any observed effect.

### Aims

Further study into the socioeconomic correlates of ED features is needed to redress the above gaps in investigation and methodological limitations of previous research. Thus, the goal of this study was to ascertain the socio-geographic and socioeconomic distribution of disordered eating, using a population-based sample of adults in South Australia. In particular, this study assessed the relative prevalence of objective binge eating, subjective binge eating, purging, strict dieting and overvaluation of weight/shape across indicators of SES, namely household income, employment status, educational level, indigenous status and urbanicity. It was hypothesised that disordered eating behaviours would be equally represented amongst all geographic areas and levels of SES.

## Methods

### Study Design and Participant Sampling

Data for this study were collected from the merged 2008 and 2009 cross-sectional Health Omnibus Survey. The survey, conducted annually by Harrison Health Research under the auspices of the South Australian Health Commission, comprises face-to-face interviews of a representative sample of the population of South Australia and includes questions related to health and demographics.

In both years, metropolitan and rural “collector districts” were selected based on a probability proportional to size sampling procedure according to the Australian Bureau of Statistics 2006 Census data. Ten houses within each district were chosen in which to conduct interviews. A total of 5000 households were selected in 2008 and 5200 in 2009. The resident who had their birthday most recently, provided they were also aged over fifteen years, was interviewed. Up to six visits were made to each household. The samples were non-replacement. To ensure participant understanding and feasibility of questions, over fifty interviews were conducted in pilot periods during January 2008 and August 2009. The survey interviews were then conducted from February to July in 2008 and from September to December in 2009. In total over the two years, 6041 people were interviewed (3034 in 2008 and 3007 in 2009). Response rates were 62.8% in 2008 and 59.3% in 2009. The most cited reason for not participating was refusal.

### Ethics

Adult participants provided verbal rather than written informed consent, due to the practicalities of carrying out a large-scale survey and the low risk nature of the survey content. Participant consent was not recorded as consent was implied through participation; participants who did not provide consent were not surveyed. For minors (15–17 year olds) enrolled in the study, written consent was obtained from the participant’s parent/guardian. This consent procedure and the survey itself were approved by the research ethics committee of the Government of South Australia, Department of Health.

### Measures

#### Socioeconomic status

Five variables were used as indicators of SES. These included household income, educational level, employment status, indigenous status, and geographic location. The exact wording of the questions used to ascertain this information is included in S1 Appendix. Household income was determined by asking participants to nominate their average annual household income bracket from the following: less than $12 000, $12 001 - $20 000, $20 001 - $30 000, $30 001 - $40 000, $40 001 - $50 000, $50 001 - $60 000, $60 001 - $80 000, $80 001 - $100 000, or greater than or equal to $100 000. Educational level was determined by asking participants to nominate their highest level of educational attainment from the following categories: still in school, left school before age 15, left school after age 15, left school after age 15 but still studying, trade qualification, certificate (less than or equal to one year completed), certificate (greater than one year completed), or bachelor degree or higher. Employment status was determined by asking participants to nominate their current employment status from the following categories: full-time work, part-time work, home duties, unemployed, retired, student, not working due to disability, or other. Indigenous status was determined by asking participants whether they identified as Aboriginal, Torres Strait Islander, both or neither. They were also given the option not to respond. Finally, geographic location was determined by asking participants to provide their postcode.

#### Eating disorder features

The survey questions aimed at eliciting information on ED features were based on diagnostic questions obtained from The Eating Disorder Examination (EDE) [28], the gold standard clinical assessment schedule for EDs. The full EDE interview was not administered due to time and financial constraints. Furthermore, as this is primarily an epidemiological study, we were interested in collecting prevalence data and the items sourced from the EDE suffice in this regard. These adapted questions have been used in the Health Omnibus Survey since 1995. The ED features assessed were objective binge eating, subjective binge eating, purging, strict dieting and overvaluation of weight/shape. In regards to behaviours, participants were asked whether they occurred regularly (at least weekly) over the previous three months. The exact wording of the questions are included in S2 Appendix. The presence of objective binge eating was assessed by asking whether participants regularly ate “an unusually large amount of food” with an accompanying sense of being “out of control”. The presence of subjective binge eating was assessed by asking whether participants regularly ate an amount of food that was not unusually large but was accompanied by the same sense of being “out of control”. The presence of purging was assessed by asking participants whether they regularly used laxatives, diuretics or self-induced vomiting as a means by which to control their weight or shape. Strict dieting was defined as regularly going on a “very strict diet” or “hardly eating anything at all” to control weight or shape. Finally, overvaluation of weight/shape was measured by having participants rate the importance they placed on weight or shape in determining their self-evaluation, from 0 (no importance) to 6 (supreme importance). A score of 5 or 6 was used to indicate the presence of overvaluation of weight/shape.

### Data Transformation and Analysis

Data from the two surveys, which comprised distinct samples interviewed 12 months apart, were merged for the purpose of the current study, as has been done previously [29]. Data in both years were weighted by the inverse of the individual's probability of selection, then re-weighted to benchmarks derived from the Estimated Resident Populations at 30th June 1994, by age, sex and Local Government Area, from the Australian Bureau of Statistics (Catalogue No 3204.4). Weighted data differed from non-weighted data. The weighted data and results are reported in this paper.

Postcode data were transformed manually into Accessibility/Remoteness Index of Australia Plus (ARIA+) scores. These scores range from 0 to 15, and provide an indication of distance by road to population centres of various sizes. Higher scores indicate less accessibility and a greater degree of remoteness. For the purposes of data analysis, scores of 6 to 15, which represent a high degree of remoteness, were combined into a single category due to low numbers for each of these scores. Likewise, multiple income brackets were grouped to form three categories (less than $30 000, $30 001 - $60 000, and greater than $60 000) and educational levels were grouped to form four categories (no tertiary qualification, trade or certificate, and university graduate).

The only variable with significant number of missing values (*n* = 1,092) was income. The missing values were imputed using a multinomial logistic regression with age, sex, marital status, work status, Australian born status and ARIA+ scores used as predictors. Results with the imputation were not significantly different from results obtained by creating a “missing” category for income. Descriptive statistics (means and standard deviations of continuous variables, number and proportions of categorical/ordinal variables) were calculated to describe the sample characteristics in terms both of demographic features and the prevalence of ED features. Five multivariate logistic regressions were employed to assess the relationship between the five ED features and the indicators of SES (income, education, ARIA+ score, indigenous status), controlling for age, sex and marital status. All tests were considered significant at the *p* < 0.05 level. All analyses were conducted using SPSS version 22.0 and *R* version 3.2.1.

## Results

### Participant Demographic Characteristics

Table 1 displays the demographic characteristics of the current sample. On average most participants were overweight or obese, middle-aged, married or in a de facto relationship, and employed either part- or full-time.

**Table 1:** Participant Demographics.

	Total Sample (*N* = 6041)^1^
Sex, *n* (%)	
Male	2960 (49.0)
Female	3081 (51.0)
Age, *M* (*SD*)	45.6 (18.9)
BMI^2^ category, *n* (%)	
Underweight (< 18.50 kg/m^2^)	94 (1.7)
Normal (18.50–24.99 kg/m^2^)	2282 (42.0)
Overweight (25.00–29.99 kg/m^2^)	1931 (35.5)
Obese (≥ 30.00 kg/m^2^)	1124 (20.7)
Marital status, *n* (%)	
Married / de facto	3681 (62.7)
Separated / divorced	521 (8.6)
Widowed	333 (5.5)
Never married	1399 (23.2)
Household income, *n* (%)	
Less than $30 000	1182 (24.8)
$30 001 - $60 000	1194 (25.1)
Greater than $60 000	2374 (50.1)
Educational level, *n* (%)	
In school	325 (5.4)
Left school / still studying	2395 (39.7)
Trade or certificate	2165 (35.9)
Bachelor’s degree or higher	1147 (19.0)
Employment status, *n* (%)	
Full-time	2287 (37.9)
Part-time	1138 (18.9)
Home duties	459 (7.6)
Student	506 (8.4)
Unemployed	125 (2.1)
Retired	1202 (19.9)
Other	320 (5.3)
Indigenous status, *n* (%)	
Aboriginal	113 (1.9)
Torres Strait Islander	4 (0.1)
Both	1 (0.0)
Neither	4367 (74.2)
Prefer not to answer	1156 (25.8)
ARIA+ score^3^, *n* (%)	
0 (high accessibility)	4587 (76.0)
1	262 (4.3)
2	315 (5.2)
3	301 (5.0)
4	98 (1.6)
5	139 (2.3)
6–15 (high remoteness)	335 (5.6)

1. Descriptive statistics are based on the number of participants who provided data. *n* = 609 participants did not report weight and/or height data, and thus BMI could not be computed for these participants; *n* = 8 participants did not report marital status; *n* = 1280 participants did not report household income; *n* = 9 participants did not report educational level; *n* = 4 participants did not report employment status; invalid postcodes were entered into the dataset for *n* = 3 participants, and thus ARIA+ scores could not be calculated for these participants.

2. BMI = body mass index

3. ARIA+ = Accessibility/Remoteness Index of Australia Plus. These scores range from 0 to 15, and provide an indication of distance by road to population centres of various sizes. Higher scores indicate less accessibility and a greater degree of remoteness.

### Prevalence of Eating Disorder Features

Table 2 displays the prevalence of the five ED features that were assessed. Overvaluation of weight/shape was highly endorsed in comparison to behavioural features. Objective binge eating was the most prevalent behaviour.

**Table 2:** Prevalence of Eating Disorder Features.

Eating Disorder Feature	Total Sample (*N* = 6041)
Objective binge eating, *n* (%)	378 (6.3)
Subjective binge eating, *n* (%)	153 (2.5)
Purging, *n* (%)	54 (0.9)
Strict dieting, *n* (%)	237 (3.9)
Overvaluation of weight/shape, *n* (%)	1166 (19.4)

NB: *n* = 15 participants did not respond to the question assessing for objective binge eating; *n* = 24 did not respond to the subjective binge eating question; *n* = 10 did not respond to the purging question; *n* = 15 did not respond to the strict dieting question; *n* = 44 did not respond to the overvaluation of weight/shape question.

### Eating Disorder Features and Socioeconomic Status

#### Household income

The odds of reporting ED features across household income levels are presented in Table 3. These regressions examined the relative likelihood of reporting ED features across income levels, relative to those with an income of less than $30 000 per year. As can be seen, no significant association with household income was found for any of the measured ED features.

**Table 3:** Effect of Income Level on Reporting of Eating Disorder Features.

	Objective Binge Eating	Subjective Binge Eating	Purging	Strict Dieting	Overvaluation of Weight/Shape
Household Income	Odds Ratio^1^ (95% Confidence Interval)				
30-60K	0.97 (0.7, 1.34)	0.79 (0.47, 1.31)	0.80 (0.37, 1.70)	1.01 (0.65, 1.56)	1.02 (0.83, 1.24)
≥60K	1.03 (0.73, 1.46)	1.15 (0.69, 1.92)	0.65 (0.28, 1.51)	1.07 (0.68, 1.70)	1.10 (0.89, 1.36)

1. Odds ratios are relative to the lowest income level (less than $30 000); *p < .05, ** *p* < .01, *** *p* < .001

#### Educational attainment

Table 4 displays the odds of reporting ED features across levels of educational attainment relative to people with the lowest educational level, namely those without tertiary qualifications. As can be seen, there was a significantly increased risk of reporting strict dieting in participants with a trade or certificate qualification. No other odds ratios were significant, indicating that educational levels do not impact the likelihood of experiencing ED features.

**Table 4:** Effect of Educational Level on Reporting of Eating Disorder Features.

	Objective Binge Eating	Subjective Binge Eating	Purging	Strict Dieting	Overvaluation of Weight/Shape
Highest Education Achieved	Odds Ratio^1^ (95% Confidence Interval)				
Trade/certificate	1.17 (0.91, 1.51)	0.92 (0.62, 1.36)	1.41 (0.80, 2.49)	1.58** (1.14, 2.18)	1.01 (0.86, 1.18)
University graduate	0.77 (0.54, 1.08)	0.98 (0.60, 1.58)	0.46 (0.15, 1.17)	1.11 (0.70, 1.75)	0.96 (0.79, 1.16)

1. Odds ratios are relative to the lowest educational level (still in school)

*p < .05

** *p* < .01

*** *p* < .001

#### Employment status

The odds of reporting ED features depending on employment status were calculated relative to those employed full-time. As shown in Table 5, participants who indicated that they were not working due to disability had a significantly greater risk of reporting objective binge eating and purging. A trend was also observed for these participants to be more likely to report strict dieting and overvaluation of weight/shape, however these estimates approached but did not reach significance. Participants who indicated they engaged in home-based duties also had a significantly greater risk of reporting overvaluation of weight/shape, and unemployed participants were at a significantly greater risk for reporting objective and subjective binge eating. Interestingly, students were at a decreased risk for reporting strict dieting. Of note, all other odds ratios were not significant, indicating a similar prevalence of ED features across levels of employment status.

**Table 5:** Effect of Current Employment Status on Reporting of Eating Disorder Features.

	Objective Binge Eating	Subjective Binge Eating	Purging	Strict Dieting	Overvaluation of Weight/Shape
Employment Status	Odds Ratio^1^ (95% Confidence Interval)				
Part time	1.29 (0.95, 1.75)	1.29 (0.80, 2.07)	0.90 (0.42, 1.94)	0.92 (0.63, 1.35)	0.96 (0.79, 1.17)
Home duties	1.09 (0.69, 1.69)	1.28 (0.63, 2.51)	0.72 (0.21, 2.06)	1.05 (0.62, 1.75)	1.39* (1.07, 1.80)
Unemployed	2.02* (1.05, 3.67)	2.80* (1.12, 6.30)	0.74 (0.04, 4.13)	1.39 (0.54, 3.11)	1.54 (0.96, 2.43)
Retired	0.91 (0.52, 1.58)	1.35 (0.61, 2.90)	2.31 (0.67, 7.94)	0.52 (0.21, 1.23)	0.90 (0.66, 1.22)
Student	0.90 (0.48, 1.63)	1.35 (0.52, 3.15)	0.16 (0.01, 1.28)	0.39* (0.14, 0.91)	1.02 (0.68, 1.50)
Disabled	2.30** (1.30, 3.92)	1.92 (0.77, 4.26)	4.13* (1.27, 12.08)	2.15 (0.99, 4.29)	1.47 (0.98, 2.16)
Other	1.16 (0.56, 2.18)	1.80 (0.70, 4.01)	1.06 (0.16, 4.11)	2.03 (0.99, 3.89)	1.06 (0.69, 1.61)

1. Odds ratios are relative to full-time employment

*p < .05

** *p* < .01

*** *p* < .001

#### Indigenous status

Table 6 displays the odds of indigenous participants reporting ED features relative to non-indigenous participants. There was an equal likelihood to report ED features, regardless of identification as an Aboriginal and/or Torres Strait Islander. Interestingly, those who did not respond to this question in the interview (*n* = 1156) were significantly more likely to report overvaluation of weight/shape.

**Table 6:** Effect of Indigenous Status on Reporting of Eating Disorder Features.

	Objective Binge Eating	Subjective Binge Eating	Purging	Strict Dieting	Overvaluation of Weight/Shape
Indigenous Status	Odds Ratio^1^ (95% Confidence Interval)				
Aboriginal or Torres Strait Islander	0.99 (0.45, 1.91)	1.54 (0.52, 3.62)	1.76 (0.28, 6.27)	1.07 (0.04, 2.38)	1.21 (0.74, 1.93)
Declined to answer	0.83 (0.63, 1.09)	0.92 (0.61, 1.35)	1.21 (0.65, 2.19)	1.03 (0.72, 1.44)	1.21* (1.04, 1.41)

1. Odds ratios are relative to reporting being of non-indigenous background

*p < .05

** *p* < .01

*** *p* < .001

#### Urbanicity

Table 7 displays the odds of reporting ED features depending on urbanicity as measured by ARIA+ scores. The odds are all relative to an ARIA+ score of 0 (which indicates the highest level of accessibility). Almost all odds ratios did not reach significance, with the exception of an ARIA+ score of 2, which was associated with a lower risk of overvaluation of weight/shape.

**Table 7:** Effect of Urbanicity on Reporting of Eating Disorder Features.

	Objective Binge Eating	Subjective Binge Eating	Purging	Strict Dieting	Overvaluation of Weight/Shape
ARIA+ Score^1^	Odds Ratio^2^ (95% Confidence Interval)				
1 (most accessible)	1.15 (0.63, 1.95)	0.90 (0.31, 2.03)	0.69 (0.11, 2.34)	0.40 (0.10, 1.07)	0.92 (0.64, 1.30)
2	0.98 (0.56, 1.59)	0.91 (0.38, 1.88)	0.55 (0.09, 1.86)	1.72 (0.97, 2.89)	0.68* (0.47, 0.95)
3	1.32 (0.82, 2.04)	1.11 (0.51, 2.14)	0.22 (0.01, 1.05)	1.34 (0.73, 2.30)	1.02 (0.75, 1.37)
4	0.74 (0.22, 1.82)	0.43 (0.02, 1.98)	0.00 (0.00, 0.00)	0.64 (0.10, 2.09)	0.55 (0.27, 1.00)
5	0.99 (0.44, 1.94)	0.87 (0.21, 2.39)	0.00 (0.00, 0.00)	0.45 (0.07, 1.46)	0.83 (0.51, 1.30)
6–15 (most remote)	1.43 (0.89, 2.19)	0.66 (0.23, 1.50)	1.45 (0.49, 3.46)	1.07 (0.53, 1.93)	0.74 (0.52, 1.03)

1. ARIA+ = Accessibility/Remoteness Index of Australia Plus. These scores range from 0 to 15, and provide an indication of distance by road to population centres of various sizes. Higher scores indicate less accessibility and a greater degree of remoteness.

2. Odds ratios are relative to the most metropolitan/accessible areas (ARIA+ score of 0)

*p < .05

** *p* < .01

*** p < .001

## Discussion

In order to determine the socioeconomic and socio-geographic distribution of disordered eating, this study examined the association between indicators of SES and the endorsement of ED features. Overall, the study found rates of ED features to be comparable across indices of SES. In particular, no association between ED features and household income or indigenous status emerged. Whilst analysis of the remaining indicators of SES–educational level, employment status and urbanicity–did yield some impact on the prevalence of ED features, the overall trend still indicated that EDs were universally experienced, irrespective of SES. This study is thus further evidence against the historical view that EDs are diseases exclusive to the wealthy [9].

Regarding the prevalence of ED symptoms in this sample, overvaluation of weight/shape was particularly prevalent (almost 20% of the sample) suggesting that having extreme concerns about weight and shape is becoming very common. Rates in previous studies have been reported to be between 10.8% [30] and 18.2% [31] of the adult population. Objective binge eating was the most common of the ED behaviours (6.3%), in line with BED being the most prevalent ED in the population [6].

Household income was not shown to have any significant impact on an individual’s likelihood of reporting any of the studied ED features. Income is the most widely investigated indicator of SES in ED research and the overarching finding of recent research concords with the findings of this study [15, 16]. Some studies have reported an increased risk of EDs in persons with lower household incomes (e.g., [32]) but these are limited by having small samples that are not representative of the general population.

Regarding educational attainment, there was a significantly increased risk of reporting strict dieting amongst participants with trade or certificate qualifications. The overall trend, however, indicated that the prevalence of ED features was largely unaffected by employment status. The findings from studies investigating educational attainment vary widely with completely opposing associations having been reported [20]. Although research has yet to reach a consensus on this topic, these disparities indicate that EDs are not exclusive to any particular level of educational attainment.

Likewise, on evaluation of the association between ED features and employment status, the overall trend was that these features were reported at similar rates irrespective of employment status. However, some correlations between specific employment statuses and specific ED features were found. Participants who were not working due to disability, unemployment or engagement in home-duties were found to be more likely to report one or more ED features. Notably, not working due to disability was associated with an increased risk of both objective binge eating and purging. The risks of reporting strict dieting and overvaluation of weight/shape were also increased but the odds ratios approached but did not reach significance. Although not in keeping with our hypothesis of equal distribution, these findings challenge historical views that EDs are limited to the wealthy [9]. In explanation of the findings related to disability, in 2002, individuals with psychological or psychiatric conditions accounted for 21.5% of recipients of the Disability Support Pension (the second most common reason for receiving the pension) [33]. Thus this finding may reflect the high prevalence of mental illness related to ED pathology among those who are disabled to the point of being unable to work; as well as the level of occupational impairment associated with EDs. The elevated prevalence of overvaluation of weight/shape among those engaged in home-based duties may be somewhat artificially inflated by the higher proportion of women in this category (97.0%), although sex was controlled for in all analyses. Finally, those who indicated they were unemployed (but not disabled or engaged in home duties) were more likely to binge eat. As ample research has shown stress to be a precursor to binge eating [34, 35], this association may be indicative of attempts to cope with the financial and social stresses associated with being out of work.

Overall, the employment status findings could also be explained by the impacts of not experiencing a work environment; those who are not working are likely to feel relatively less productive and more socially isolated than those who are working which may contribute to the development of disordered eating. This theory it supported by a study by Theodossiou which found employment status to greatly impact an individual’s psychological wellbeing [36]. Unemployed participants were found to have a significantly greater risk of low self-esteem, low self-confidence, feelings of unhappiness and being unable to enjoy day-to-day activities. Low self-esteem is proposed as a core risk factor of the development of EDs [2]. This suggests that the benefits of employment to psychological wellbeing are not purely attributable to financial security and also stem from the actual experience of working.

One final, and unexpected, impact of employment status identified in this study was that students were at a decreased risk for reporting strict dieting. Although this was not predicted in our hypothesis, the finding that strict dieting is more common amongst the other groups indicates that disordered eating is not limited to the young. In fact, research has shown that older men and women are increasingly reporting strict dieting. Mitchison et al. found that, between 1998 and 2008, strict dieting increased among adults aged 25–44 years and over 45 years, but not in those aged 15–24 years [15].

This paper provides one of the only assessments of the link between indigenous status and the prevalence of ED features in the Australian population. Our findings indicate that indigenous Australians are certainly not immune from disordered eating, with rates of each feature assessed being equivalent to those of the non-indigenous sample. The results of this study mirror those of the only other study assessing the prevalence of ED features in indigenous Australians [26], which also found rates of ED features to be comparable between indigenous and non-indigenous participants. Furthermore, these findings are consistent with studies of indigenous peoples from other nations. A study of indigenous Fijians [37] found rates of binge eating and BED consistent with those reported in Western populations, despite opposing cultural attitudes and practices relating to diet and the ideal body shape and size. Together, these and other studies suggest that disordered eating can occur in a range of social and cultural contexts.

An interesting finding from the analysis of indigenous status was that those who refused to answer the question as to whether they identified as Aboriginal and/or Torres Strait Islander (25.8% of the sample) were significantly more likely to report overvaluation of weight/shape. Given that the proportion of participants in this sample who identified as Indigenous Australian (2.0%) was similar to the known proportion in the Australian population (2.4%), it is unlikely that many of the non-respondents were also indigenous. A possibility is that non-response to questions is indicative of broader insecurity, which is also experienced in relation to one’s appearance. However, this is speculative and the association found between these two factors may represent a serendipitous statistical finding.

Finally, this study found no overall association between urbanicity and ED features. Although there was a statistically significant decreased risk of overvaluation of weight/shape amongst participants with an ARIA+ score of 2, there was no discernible overall pattern and these results are unlikely to be replicable. The majority of studies investigating the association between urbanicity and ED prevalence have likewise found no association [15, 16, 38, 39]. The current study adds to the existing research by providing more precise findings by use of ARIA+ scores rather than a simple division into urban and rural which is typical of studies on this topic.

### Strengths and Limitations

The majority of studies examining EDs and SES focus on income alone. However, social disadvantage has multifactorial determinants. A strength of this study is that it analysed multiple indicators of social disadvantage. Another common limitation of research into ED demographics mentioned previously is the use of clinical samples. Such samples tend to be dominated by persons of higher SES living in urban areas (who have easier access to these services) with AN or BN, resulting in a skewed picture. This study, in contrast, was community-based, and thus we have greater confidence in the generalisability of the findings to the broader population of adults with EDs. Many studies examining SES used simple division of variables into only two groups such as high versus low income or urban versus rural residence. The use of three brackets to indicate household income and ARIA+ scores to indicate urbanicity allowed for a more precise evaluation of the association between these variables and the prevalence of ED features than has been possible in previous studies. Another strength of the study was the symptom-based approach. Persons of lower SES (defined by the indicators in this study) face additional barriers to ED detection and diagnosis such as not being able to afford mental health services, living in rural or remote regions where such services are not readily available, or not being considered likely to have an ED due to not fitting the demographic stereotype. As such, it is possible that diagnosis rates in such groups are lower and a symptom-based approach is thus a more valid method of measuring the prevalence of disordered eating than the use of ED diagnoses. Finally, this study had a large sample size and included participants of both sexes and a wide range of age groups.

Limitations are also observed in the methodology of the current study. Despite the large sample size, the number of indigenous participants and participants who reported purging were small, which reduced statistical power in detecting differences in these groups. Additional limitations included that the surveys did not include questions to assess for the presence of excessive exercise or body composition concerns (typically a desire for increased muscularity) which are important ED features, particularly in males. Likewise, measures of depressive or anxiety symptoms were not included due to feasibility constraints. As mood, anxiety and other mental disorders commonly co-exist with EDs [6], such measures would have been useful covariates. However, the absence of these questions did not hinder the main purpose of this study which was to assess the association between ED features and SES. Next, ED features were identified by use of structured interviews by laypersons rather than clinical interview by an expert clinician. Finally, South Australia is a largely metropolitan state and thus may not be the ideal population for a study assessing the geographic distribution of ED.

### Clinical and Public Health Implications

The finding that disordered eating is equally prevalent irrespective of SES has significant implications for the provision of ED treatment. Specialist ED services are typically concentrated in large metropolitan areas and typically located in the more affluent areas. Furthermore, many of these services are within the private healthcare sector. Naturally, the targeting of treatment and support services to such a specific subset of the population limits access to persons not meeting the traditional ‘young wealthy female’ stereotype. For these reasons, specialist services are less accessible to persons of lower SES. As such, it would be beneficial to provide more public ED services as well as having services distributed throughout these large metropolitan areas, not just the more affluent areas. In rural and remote communities, access to ED services is even more difficult as there is a distinct lack of health practitioners with ED expertise in these areas. Potential strategies to improve access in these areas include training local healthcare workers in the detection and treatment of EDs and delivering treatment through telemedicine. A study comparing cognitive behavioural therapy (CBT) in BN delivered in person versus via telemedicine found that CBT via telemedicine had comparable outcomes to face-to-face CBT and was readily accepted by patients [40]. Finally, as this study and other research [26, 37] have shown disordered eating behaviours to occur at similar rates amongst indigenous and non-indigenous persons, it is vital that culturally sensitive ED services with staff who have been trained and are experienced in the treatment of indigenous persons are made available, especially considering the high burden of obesity and weight-related health problems in this population.

Changes also need to be made to the practice of individual healthcare workers as evidence has shown that healthcare workers might be biased towards the historical stereotype. As mentioned previously, Becker et al. found that non-White women are less likely to be asked about ED features by their general practitioner than White women [12]. It is thus vital that general practitioners and other healthcare workers consider EDs in persons of any demographic group–and this should be evident in the teaching curricula of medicine and allied health students. Similarly, based on the findings of the current study, general practitioners who practice in or see patients from rural or lower socioeconomic communities should be educated in the detection and referral of EDs among these groups.

Finally, the targeting of treatment also contributes to stigma that discourages non-traditional patients from seeking treatment [41]. For instance, males seek treatment for BED and BN at approximately 60% the rate of females [12]. This disparity is largely attributed to a fear of being viewed as being “less of a man” for having a condition traditionally viewed as largely affecting women [41]. Furthermore, stigmatisation has also been shown to be associated with a longer duration of illness, lower self-esteem and higher levels of comorbid mental illness. It is thus vital that the public perception of EDs occurring only in young women be corrected. One method of doing so would be ensuring that public health campaigns are directed at and include people with EDs of diverse backgrounds (e.g. males, people of non-White ethnicity, and older people).

While ED features overall were shown to be equally prevalent irrespective of employment status, not working due to disability, engagement in home duties or unemployment were found to be associated with an increased risk of specific ED features. A possible contributor to this may be social isolation and lack of productivity stemming from not being in a work environment. As such, providing support to such people to potentially enable them to join the work force, or encouraging them to engage in other social or community activities that may provide a sense of self-worth and life satisfaction could reduce the burden of disordered eating in these groups.

### Future Research

The results of this study open up potential areas for future research. Firstly, low SES is generally associated with under-detection and diagnosis of medical conditions. It would thus be interesting to assess the relationship between SES and an identified ED diagnosis. This could be achieved by adding a question in future similar surveys about whether participants had ever received a diagnosis of an ED from a doctor or other health care practitioner. Moreover, further questions could be added to more accurately determine diagnostic status according to DSM criteria [1]. Next, to address the issue of general practitioners not considering EDs amongst all patients, two more potential areas for research would include surveying clinicians in public health and rural areas to identify their gaps in knowledge and service provision, and piloting an intervention to improve general practitioner confidence and accuracy in detecting EDs.

### Conclusion

The finding that disordered eating is equally prevalent irrespective of SES has significant implications for the provision of ED treatment. There needs to be a move away from targeting services toward a particular subset of the population so that all sufferers can have equitable access to treatment. The provision of services in rural and remote regions–whether it by as local services or outreach programs from city hospitals–as well as metropolitan areas of low SES is particularly pertinent. Further, doctors and other healthcare workers are advised to consider eating disorders in persons of any demographic group. Finally, although unexpected, the finding that various ED features are more prevalent amongst those who are not working is of great import as it highlights a potential target to reduce the burden of disordered eating in the community. Providing support to assist such people in joining the work force, or encouraging their engagement in meaningful social or community activities could improve resilience against the development of eating disorders.

## Supporting Information

S1 Appendix Health Omnibus Survey Questions (Participant Demographics). (DOCX)

S2 Appendix Health Omnibus Survey Questions (Eating Disorder Features). (DOCX)

## Abbreviations

ARIA+: Accessibility/Remoteness Index of Australia Plus
AN: anorexia nervosa
BED: binge eating disorder
BN: bulimia nervosa
CBT: cognitive behavioural therapy
ED: eating disorder
EDE: The Eating Disorder Examination
OSFED: other specified feeding and eating disorders
SES: socioeconomic status
UFED: unspecified feeding and eating disorders
