# Supplementary material for: Socioeconomic Correlates of Eating Disorder Symptoms in an Australian Population-Based Sample
Source: PLoS One. 2017 Jan 31;12(1):e0170603. doi: 10.1371/journal.pone.0170603 (PMC5283666; doi:10.1371/journal.pone.0170603)
Supplement: S1 Appendix — (DOCX) [file pone.0170603.s001.docx]

**S1 Appendix: Health Omnibus Survey Questions (Participant Demographics)**

| 1. classifications |  |
| --- | --- |
| - 1. Are you of Aboriginal or Torres Strait Islander Origin?  1. No 2. Aboriginal 3. Torres Strait Islander 4. Both 5. Don’t know |  |
| - 1. Which of these groups best describes the highest qualification you have obtained? *Show prompt card Z.2.*  1. **Still at school** (Go to Z.7) 2. **Left school at 15 years or less** 3. **Left school after age 15** 4. **Left school after age 15 but still studying** 5. **Trade qualification/apprenticeship** 6. **Certificate/Diploma – one year full time or less** 7. **Certificate/Diploma – more than one year full time** 8. **Bachelor degree or higher** |  |
| - 1. Which of these best describes your work status? Record age in years  1. **Work full time** 2. **Work part time** 3. **Home Duties** 4. **Unemployed** 5. **Retired** 6. **Student** 7. **Other** 8. **Not working because of work related injury** |  |
| - 1. I would now like to ask you about your household’s income. We are interested in how income relates to lifestyle and access to health services. Before tax is taken out, which of the following ranges best describes your household’s income, from all sources, over the last 12 months? *Show prompt card Z.3.*  1. **Up to $12,000** 2. **$12,001 - $20,000** 3. **$20,001 - $30,000** 4. **$30,001 - $40,000** 5. **$40,001 - $50,000** 6. **$50,001 - $60,000** 7. **$60,001 - $80,000** 8. **$80,001 - $100,000** 9. **$100,000 or more** 10. **Not stated** |  |
| - 1. What is your postcode?   *Enter postcode* |  |
