# Supplementary material for: Socioeconomic Correlates of Eating Disorder Symptoms in an Australian Population-Based Sample
Source: PLoS One. 2017 Jan 31;12(1):e0170603. doi: 10.1371/journal.pone.0170603 (PMC5283666; doi:10.1371/journal.pone.0170603)
Supplement: S2 Appendix — (DOCX) [file pone.0170603.s002.docx]

**S2 Appendix: Health Omnibus Survey Questions (Eating Disorder Features)**

| 1. weight control |  |
| --- | --- |
| I would now like to ask you about episodes of overeating. By overeating, or binge eating, I mean eating an unusually large amount of food in one go and at the time feeling that your eating was out of control. Interviewer note respondent could not prevent themselves from overeating, or could not stop eating once they had started.   - 1. Over the past three months how often have you overeaten? Would you say…  1. **Not at all** 2. **Less than weekly** 3. **Once a week** 4. **Two or more times a week** 5. Don’ t know 6. Refused |  |
| - 1. Over the past 3 months have you felt your eating was out of control when others might not agree the amount of food was unusually large (e.g. 2-3 pieces of bread)? Would you say…. Interviewer note: the respondent could not prevent themselves from overeating, or they could not stop eating once they had started on smaller or more usual amounts of food.  1. **Not at all** 2. **Less than weekly** 3. **Once a week** 4. **Two or more times a week** 5. Don’ t know 6. Refused |  |
| The next questions are about various weight-control methods some people use.   - 1. Over the past three months have you regularly used, that is at least once a week, any of the following: laxatives, diuretics (water tablets), made yourself sick, in order to control your shape or weight?  1. Yes 2. No 3. Refused |  |
| - 1. Over the past three months have you regularly done any of the following: gone on a very strict diet, or eaten hardly anything at all for a time, in order to control your shape or weight? Interview note: at least once weekly, or recurrently during the three months  1. Yes 2. No 3. Refused |  |
| - 1. On a scale of 0-6, where 0 is Not at all important and 6 is Extremely important how important has your weight and/or your shape influenced how you think about (judge) yourself as a person in the past three months ? Interviewer note: has it been a really important issue to them or their self-confidence?   Record number or circle **R** for refused | **R** |
